# Supplementary material for: Magnesium Hydroxide Nanoparticles Kill Exponentially Growing and Persister Escherichia coli Cells by Causing Physical Damage
Source: Nanomaterials (Basel). 2021 Jun 16;11(6):1584. doi: 10.3390/nano11061584 (PMC8234494; doi:10.3390/nano11061584)
Supplement: Supplementary file 1 [file nanomaterials-11-01584-s001.zip › nanomaterials-1232287-supplementary.pdf]

SUPPLEMENTARY MATERIALS

Article

Magnesium Hydroxide Nanoparticles Kill Exponentially Growing and Persister *Escherichia coli* Cells by Causing Physical Damage

Yohei Nakamura [1], Kaede Okita [1], Daisuke Kudo [2], Dao Nguyen Duy Phuong [2], Yoshihito Iwamoto [2], Yoshie Yoshioka [1], Wataru Ariyoshi [1] and Ryota Yamasaki [1,*]

[1] Department of Health Promotion, Division of Infections and Molecular Biology, Kyushu Dental University, Kitakyushu 803-8580, Fukuoka, Japan; r19nakamura@fa.kyu-dent.ac.jp (Y.N.); r19okita@fa.kyu-dent.ac.jp (K.O.); r16yoshioka@fa.kyu-dent.ac.jp (Y.Y.); arikichi@kyu-dent.ac.jp (W.A.)

[2] Kyowa Chemical Industry Co., Ltd., Hayashida-cho, Sakaide 762-0012, Kagawa, Japan; daisukekudo@kyowa-chem.co.jp (D.K.); daonguyenduyphuong@kyowa-chem.co.jp (D.N.D.P.); yoshihitoiwamoto@kyowa-chem.co.jp (Y.I.)

* Correspondence: r18yamasaki@fa.kyu-dent.ac.jp; Tel.: +81-93-285-3051

Table S1. Data of XRD measurements. FWHM (full width at half maximum) indicates diffraction line width spread.

	**FWHM** [°2Th.]	**Crystalline size** [A]	**Crystal plane**
	0.830	97	(001)
**NM80**	0.520	161	(101)
	0.190	479	(110)
	0.235	342	(001)
**NM300**	0.199	423	(101)
	0.150	607	(110)
	0.165	489	(001)
**NM700**	0.163	515	(101)
	0.155	587	(110)

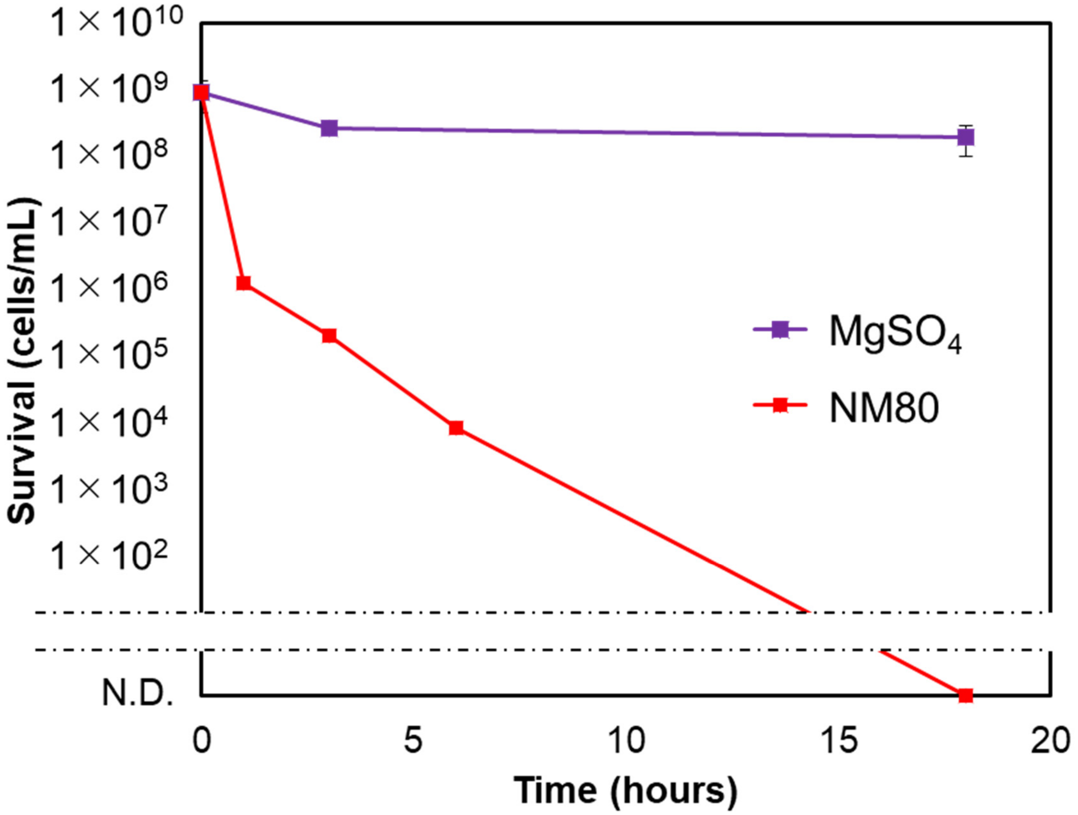

Figure S1. Bactericidal effects of $MgSO_4$ on *E. coli* at a concentration of 500 mg/l (purple line).

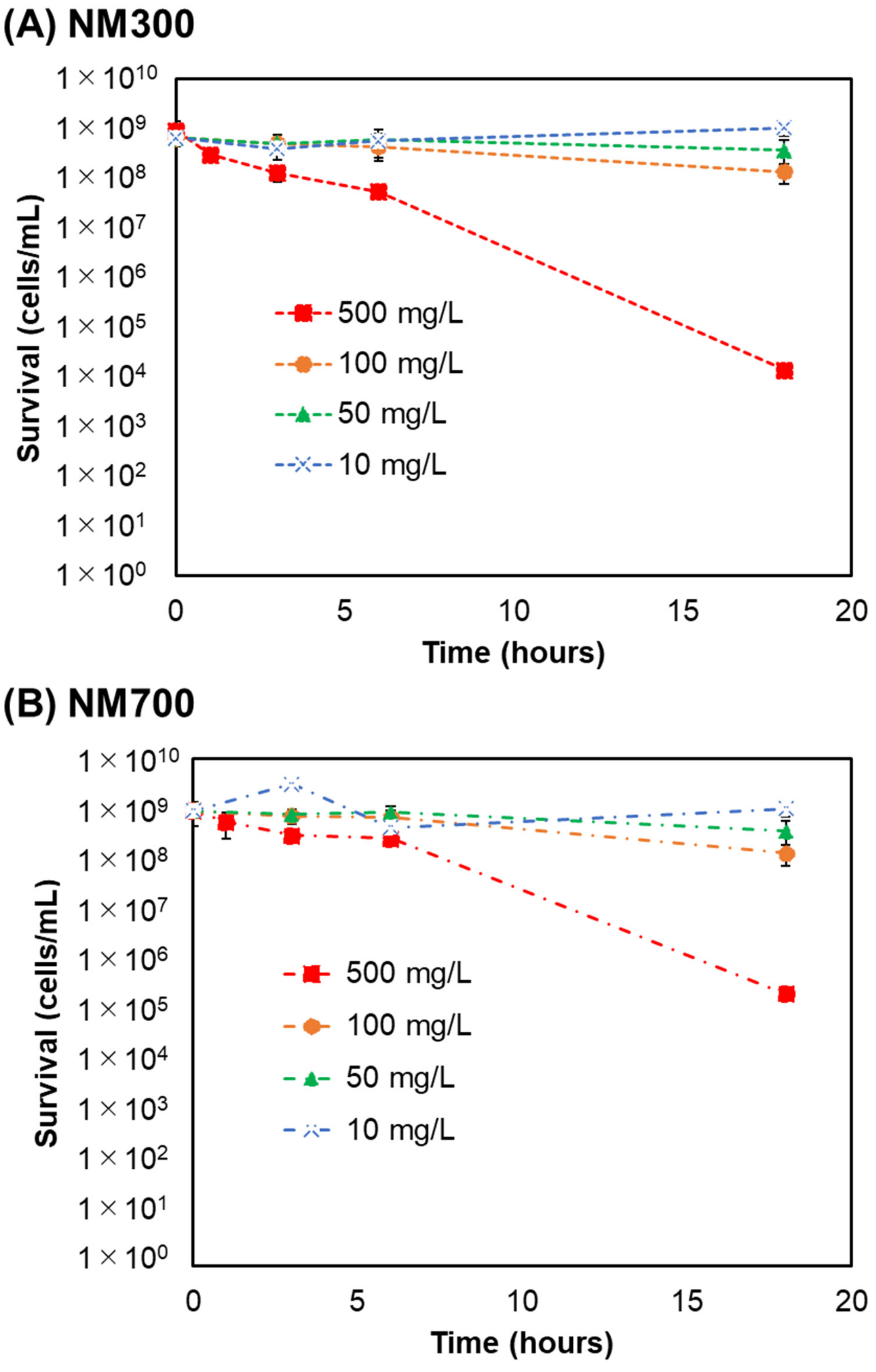

Figure S2. Bactericidal effect of NM300 (**A**) and NM700 (**B**) at different concentrations; 500 mg/l (red), 100 mg/l (orange), 50 mg/l (green), and 10 (blue) mg/l were examined for sterilizing *E. coli*.

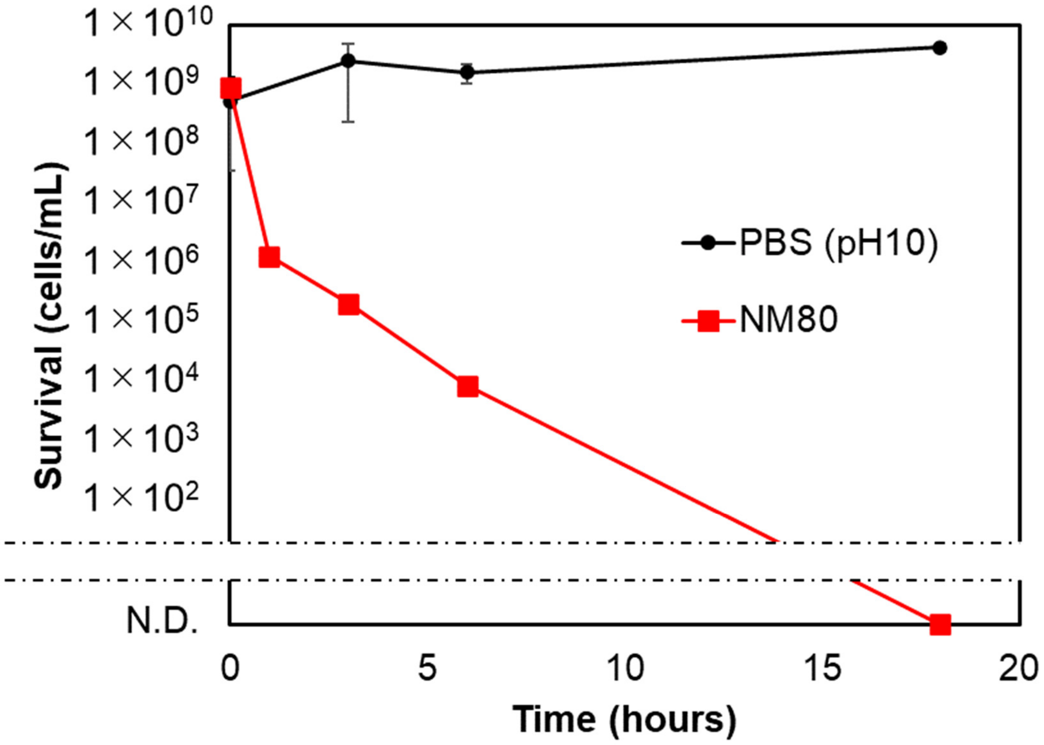

Figure S3. Bactericidal effects of PBS on *E. coli* at pH 10 (black line).

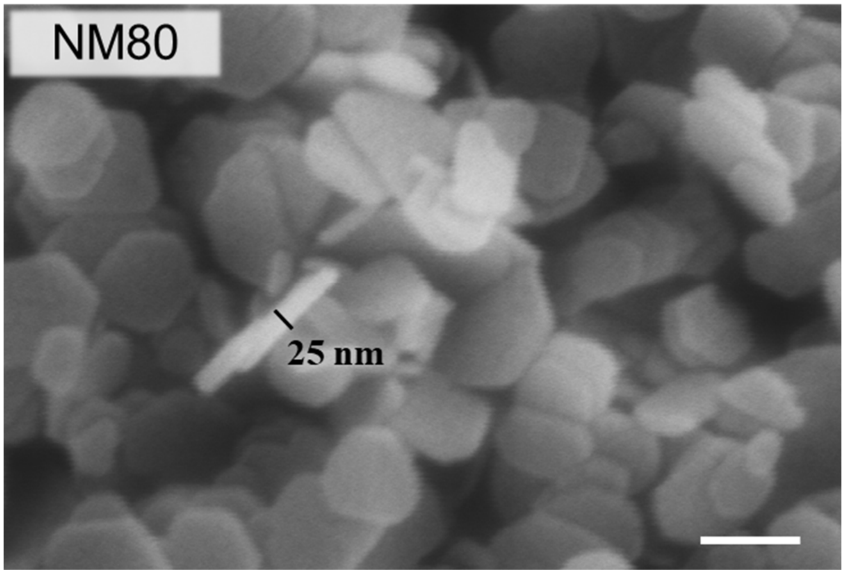

Figure S4. Magnified SEM images of NM80. Scale bar indicates 100 μm. Black line indicates the thickness of NM80.
